# Prevalence and determinants of *Campylobacter* infection among under five children with acute watery diarrhea in Mwanza, North Tanzania

**DOI:** 10.1186/2049-3258-72-17

**Published:** 2014-05-30

**Authors:** Anna-Pendo Deogratias, Martha F Mushi, Laurent Paterno, Dennis Tappe, Jeremiah Seni, Rogatus Kabymera, Benson R Kidenya, Stephen E Mshana

**Affiliations:** 1 Department of Pediatrics and Child health, Catholic University of Health and Allied Sciences, Mwanza, Tanzania; 2 Department of Microbiology/Immunology, Catholic University of Health and Allied Sciences, Mwanza, Tanzania; 3 Institute of Hygiene and Microbiology, University of Wuerzburg, Wuerzburg, Germany; 4 Department of Biochemistry and Molecular Biology, Catholic University of Health and Allied Sciences, Mwanza, Tanzania

**Keywords:** Acute watery diarrhea, Campylobacteriosis, Under five children

## Abstract

**Background:**

Campylobacteriosis, a zoonotic bacterial disease observed world-wide, is becoming the most commonly recognized cause of bacterial gastroenteritis in humans. This study was done to determine the prevalence and determinants of *Campylobacter* infection among under-fives with acute watery diarrhea in Mwanza City, Tanzania.

**Method:**

This cross-sectional hospital-based study was conducted at Bugando Medical Centre (BMC) and Sekou Toure Hospital in Mwanza City. All inpatients and outpatients under-fives who met the inclusion criteria from October 2012 to April 2013 were enrolled in the study. Demographic and clinical data were obtained using standardized data collection tools. Stool samples were collected for gram staining and culture for *Campylobacter* spp. on Preston selective agar media. In addition, blood slides for malaria and HIV tests were done to all patients.

**Results:**

A total of 300 children were enrolled with a median age of 12 [interquartile range, 8–19] months. Of these, 169 (56.5%) were from BMC and 131 (43.7%) from Sekou-Toure hospital. One hundred and seventy (56.7%) of the participating children were male. Of 300 under-fives with acute watery diarrhea, 29 patients (9.7%) were found to have *Campylobacter* infection. A significant higher number of children with *Campylobacter* infection were found in Sekou Toure hospital compared to BMC [16.0% (21/29) versus 4.7% (8/29), *p* = 0.002)]. Age above 2 years was independently found to predict campylobacter infection (OR: 2.9, 95% CI 1.1-7.7, *p* = 0.0037). Of 30 patients with a positive blood slide for *Plasmodium falciparum*, 20.0% were also positive for *Campylobacter* infection (OR: 3.9, 95% CI 1.2-10.1, *p* = 0.021).

**Conclusion:**

*Campylobacter* infection shows a comparatively low prevalence in under-fives with acute watery diarrhea in Mwanza city and is independently associated with positive slides for malaria and an age above 2 years. Further studies are needed to type the most prevalent *Campylobacter* species and to determine their antibiotic susceptibility pattern.

## Background

Diarrhea remains the most common problem affecting under-fives in developing countries [1]. Prevalence of *Campylobacter* infections in developing countries is estimated to be higher than that of *Salmonella* and *Shigella*[2]. The disease is rapidly becoming the most commonly recognized cause of bacterial gastroenteritis in human and is estimated to cause 5–14% of the cases of diarrhea worldwide [3].

*Campylobacter* enteritis is usually self-limiting with gradual improvement in symptoms over several days, but in approximately 10%-20% of cases are associated with prolonged or severe illness [3]. Asymptomatic infection is also more common in developing countries than in industrialized countries [1]. In Bangladesh, for example, up to 39% of all children younger than 2 years have an asymptomatic *Campylobacter* infection [1]. A previous study in Tanzania showed a prevalence of 4% of *Campylobacter* infection in asymptomatic patients aged <18 months [4].

Poor hygiene, sanitation and close proximity of man and animals in developing countries facilitate a frequent acquisition of enteric pathogens including *Campylobacter* spp; these factors are responsible for high prevalence of the disease in these countries [4,5]. *Campylobacter* infections have shown seasonality in occurrence. A study in Egypt showed that episodes of *Campylobacter*-associated diarrhea were detected with consistently higher rates during the warmer months, between May and August [5]. Despite the high prevalence of diarrhea in our setting, there is paucity of data regarding the epidemiology of *Campylobacter* infection among children with acute watery diarrhea. This study therefore aimed at determining the magnitude and determinants of *Campylobacter* infection among under-fives attending the Bugando Medical Centre (BMC) and Sekou Toure hospital in Mwanza City, Tanzania.

## Methods

### Study design

This was a hospital based cross-sectional study conducted at BMC and Sekou-Toure hospital. The BMC is a tertiary University referral hospital in northwestern of Tanzania, while Sekou Toure Hospital is a regional hospital in Mwanza City.

### Study population and sample size

Children aged between 1 to 60 months with acute watery diarrhea were eligible to participate in the study. Acute watery diarrhea was defined as; any child with 3 or more abnormally loose or fluid stools in the past 24 hours with or without dehydration (http://whqlibdoc.who.int/publications/2005/9241593180.pdf). The minimum sample size of 227 was estimated using the Kish Lisle formula [6] using a previous prevalence of 18% [4]. To increase the power of the study, a total of 300 under-fives were enrolled. All children who met the inclusion criteria presenting at the two hospitals were serially requested to participate in the study until the sample size was reached. Written informed consent from the parents was obtained. Standardized data collection tool was used to collect demographic and clinical data. The research protocol was approved by the Joint BMC/CUHAS ethics committee (CREC/004/2013).

### Specimen collection and laboratory procedures

Stool specimens were collected on the day of enrollment with sterile containers (HiMedia, India), and sent to the CUHAS microbiology laboratory immediately. Two microscopic smears were prepared from each stool specimen. Slides were stained with 1% carbol fuchsin and with 0.3% carbol fuchsin as gram counter stain, respectively [7]. Mucoid areas of the stool were inoculated in Preston selective agar media (Oxoid, UK) and incubated at 42°C for 48 hours under microaerophilic conditions generated by gas packs (CAMPY GEN,OXOID LTD UK) [7].

A known *Campylobacter* isolate from chicken feces was used as a positive control to assure the quality of the media, reagents and incubation conditions. *Pseudomonas aeruginosa* ATCC 9027 was used as positive control for oxidase testing and *Staphylococcus aureus* ATCC 25923 for catalase testing. All slides were examined by two individuals independently under light microscope using × 10 magnification for white blood cells detection and × 100 magnification oil immersions for *Campylobacter* visualization. Results were verified independently by two clinical microbiologists before culture results were known. In case of disagreement a third microbiologist was consulted.

Giemsa stained blood slides for malaria parasites were analyzed as described previously [8]. All slides were read by 2 experienced microscopists and all discordant slides and 10% of positive and negative slides which were selected randomly were examined by the third expert for quality control. HIV testing was done as per new Tanzanian national algorithm using rapid HIV tests; Determine HIV1/2 (Alere Medical Company, Japan) as first test followed by Unigold (Trinity Biotech. Bray. Ireland) as the second test [9]. For children below 18 months PCR was used [10].

### Data management and statistical analysis

Data were double entered using Microsoft Excel 2007 and analyzed using STATA version 11 (College station, Texas). Categorical variables were reported as proportion and were compared using Pearson Chi squared test or Fischer’s exact test where appropriate. Continuous data were described as means (standard deviation) or medians (inter quartile range) depending on the distribution of data. *Campylobacter* infection was defined as either positive culture or positive slide. To determine determinants of *Campylobacter* infection we used backward-stepwise logistic regression model to select factors with a p-value of less than 0.1 to be fitted into the multivariate logistic regression analysis. Odds ratios (OR) and their 95% confidence interval [95% CI] were computed. Factors with the p-value of less than 0.05 on multivariate logistic regression analysis were considered to have a statistically significant association with Campylobacter infection.

## Results

A total of 3962 children were admitted to BMC and Sekou Toure Hospital from October 2012 to April 2013; of these 1787 and 2275 were from BMC and Sekou Toure, respectively. A total of 300 children were enrolled with a median age of 12 [8-19] months. Of these, 169 (56.5%) were enrolled from BMC and 131 (43.7%) from Sekou Toure Hospital. The majority of children were males (170 children, 56.7%) and below 24 months of age (87%), (Table 1). Among 300 patients who were enrolled into the study 213 (71%) were treated with anti-malarial (artemisinins-lumefantrine) and 176 (58.6%) had used oral antibiotics (amoxicillin, co-trimoxazole or erythromycin) prior to enrollment. Blood slides for malaria parasites were positive in 30 (10%) of the children.

**Table 1:** Characteristics of study population

**Study variable**	**Number of patients**	**Percentage**
Hospital		
BMC	169	56.3
Sekou-Toure	131	43.7
Sex		
Female	130	43.3
Male	170	56.7
Age		
≤ 24 Months	261	87
>24 Months	39	13
Body temperature		
<37.5	95	31.7
*>37.5*	*205*	*68.3*
Malaria		
Positive	30	10.0
Negative	270	90.0
HIV Status		
Positive	20	6.7
Negative	280	93.3

### Prevalence and predictors of *Campylobacter* infection

Of 300 under-fives with acute water diarrhea, 29 (9.7%) were found to have an acute *Campylobacter* infection. A significant higher number of children with Campylobacter infection were found in Sekou Toure hospital compared to BMC [16.0% (21/29) versus 4.7% (8/29) (OR = 3.8; 95% CI = 1.6-9.0; *p* = 0.002). No significant association was found between using tap water, well water or river/lake water and boiling of water with *Campylobacter* infection while keeping cows had a borderline association with the disease on the univariate analysis (OR = 2.8; 95% CI = 0.9-8.1; *p* = 0.054) (Table 2).

**Table 2:** Predictors of Campylobacter infection among under five children attending at BMC and Sekou-Toure

**Risk factors**	**Campylobacter infection**		**Unadjusted**		**Adjusted**	
	**YES**	**NO**	**OR [95% CI]**	**P-value**	**OR [95% CI]**	**P-value**
	**n (%)**	**n (%)**				
***Hospital***						
BMC	8 (4.7)	161 (95.3)	1		1	
Seko Toure	21 (16.0)	110 (84.0)	3.8 [1.6-9.0]	0.002	4.0 [1.7-4.7]	0.002
***Sex***						
Male	16 (9.4)	154 (90.6)	1		-	-
Female	13 (10.0)	117 (90.0)	1.1 [0.5-2.3]	0.864	-	-
**Age**						
≤24 months	21 (8.1)	240 (91.9)	1		1	
>24 months	8 (20.5)	31 (79.5)	2.9 [1.2-7.2]	0.018	2.9 [1.1-7.7]	0.037
***Body Temperature***						
≤37.5	6 (6.3)	89 (93.7)	1			
>37.5	23 (11.0)	182 (88.0)	1.9 [0.7-4.8]	0.187	-	-
***Malaria***						
No	23 (8.5)	247 (91.5)	1		1	
Yes	6 (20.0)	24 (80.0)	2.7 [1.0-7.2]	0.051	3.4 [1.2-10.1]	0.021
***HIV Status***						
Positive	1 (5.0)	19 (95.0)	1			
Negative	28 (10.0)	252 (90.0)	2.1 [0.3-16.4]	0.475	-	-
***Boiling Water***						
Yes	15 (8.3)	165 (91.7)	1			
No	14 (11.7)	106 (88.3)	1.5 [0.7-3.1]	0.341	-	-
***Tap Water use***						
Yes	5 (7.0)	66 (92.7)				
No	24 (10.5)	24 (10.5)	1.5 [0.6-4.2]	0.395	-	-
***Well Water use***						
Yes	4(7.27)	220 (89.0)	1			
No	25(10.0)	51 (92.7)	0.7 [0.2-2.1]	0.508	-	-
***Keeping Chicken***						
No	18 (9.1)	180 (90.9)	1			
Yes	11(10.8)	91 (89.2)	1.2 [0.5-2.7]	0.639	-	-
***Keeping Cows***						
No	24 (8.7)	252 (91.3)	1		1	
YES	5 (20.8)	19 (89.2)	2.8 [0.9-8.1]	0.063	2.3 [0.7-7.5]	0.163
***Antibiotics Use***						
Yes	19 (8.0)	218 (92.0)	1		1	
No	10 (15.9)	53 (84.1)	2.3 [1.0-5.0]	0.06	1.5 [0.6-3.8]	0.340

Upon multivariate logistic regression analysis, children attending Sekou Toure where more likely to be found with campylobacter infection than children attending BMC (OR = 2.9; 95% CI = 1.1-7.7; *p* = 0.037). In addition on multivariate logistic regression analysis; age above 24 months and malaria co-infection were found to be significant predictors of *Campylobacter* infection among children with acute watery diarrhea (OR = 4.0; 95% CI = 1.7-9.7; *p* = 0.002 and OR = 3.4; 95% CI = 1.2-10.1; *p* = 0.021 respectively) (Table 2).

### Antibiotic use and campylobacter infection

Of 169 under-fives from BMC, 141 (83.4%) had used antibiotics compared to 35 (26.7%) out of 131 under-fives from Sekou Toure hospital (*p* < 0.001). Of 141 children from Bugando Medical Centre who used antibiotics 4/141 (2.8%) had campylobacter infection compared to 4/28 (14.3%) of those who did not use antibiotics (*p* = 0.019). No significant difference regarding antibiotic use and campylobacter infection was observed among children from Sekou Toure (Table 3).

**Table 3:** Sub-analysis of antibiotics use history as a predictor of Campylobacter infection by hospitals

**Hospital**	**Antibiotic use**	**Campylobacter infection**		**Unadjusted**	**P-value**
		**Yes**	**No**	**OR [95% CI]**	
		**n (%)**	**n (%)**		
**BMC**	Yes	4 (2.8)	137 (97.2)	1	
	No	4 (14.3)	24 (85.7)	5.7 [1.3 – 24.4]	0.019
**SEKOTOURE**	Yes	6 (17.1)	29 (82.9)	1	
	No	15 (15.6)	81 (84.4)	1.1 [0.4 – 3.2]	0.834

## Discussion

### General patient characteristics and clinical co-morbidities

This hospital-based study involved 300 patients below the age of 60 months with watery diarhorea. The median age was 12 months, a finding which is similar to results obtained from a study in Kampala, Uganda [11]. Similar demographic characteristics were also observed in previous studies in Mozambique and Kenya [12,13]. Diarrhea in children has shown predilection to affect children below 2 years of age more than any other age group [1]. This was confirmed also in our study, in which 74% of the recruited children were below 2 years of age.

In view of clinical co-morbidities, this study found that of 300 under-fives, 10% had concurrent malaria. The study in Mozambique had reported similar findings [12]. Similar to the study in Mozambique, about two third of children in the present study had fever [12].

### The prevalence of Campylobacter infection

This study found the proportion of under-fives with *Campylobacter* infection among children with acute watery diarrhea to be 9.7%. Similarly, studies from Uganda and the Central African Republic have shown a prevalence of 9.3% and 10.9% respectively [11,12]. However, the findings from this study differ from results obtained in a previous studies in Tanzanian, Malawi and South Africa which reported higher prevalence of 18%, 21% and 47.4% respectively [4,12,13]. The low prevalence in our study could be attributed by the prior use of antibiotics before culture. Of 300 patients enrolled in this study, 58.6% used antibiotics (amoxicillin, erythromycin or co-trimoxazole) prior to admission for treating other pediatric morbidities such as respiratory infections and urinary tract infections. At BMC, significantly higher prevalence of *Campylobacter* infections was seen in those children who had not used antibiotics compared to those who had taken antibiotics; this further supports why the prevalence is low in our study when compared to other studies. Higher prevalence of *Campylobacter* infection was observed in children recruited from Sekou Toure Hospital than from BMC (*p =* 0.002). This could be contributed by the use of antibiotics; children from Bugando Medical Centre significantly used antibiotics than those from Sekou Toure (p < 0.001). Children attending Sekou Toure come directly from their home so they are less likely to use antibiotics prior to admission while those at BMC are from other health facilities around the Lake Zone so they are likely to be given antibiotics for a significant duration before they are referred to BMC. The observed prevalence in Sekou Toure Hospital (16%) which is comparable to the previous prevalence of 18% observed in the Mwanza region nearly two decades ago [4]. These findings suggest that the magnitude of *Campylobacter* infection has not changed much in the area; this necessitates intensified strategies to control hygiene and sanitation. In previous studies higher prevalence of *Campylobacter* infection have been observed in children with acute watery diarrhea below the age of 24 months [4,11], contrary to the findings of our study which showed higher infection rates in children above 2 years of age. The reason for this difference could not be established in this study but could partly be explained by the fact that children in this study were not exposed to source of infection before therefore non immune [14], until they were old enough to move around on their own. Also this might suggests changing in epidemiology of campylobacter infection towards that of developed countries [15].

The present study was performed during the rainy season only and thus, seasonality in association to *Campylobacter* infection could not be observed. However, a previous study in Tanzania reported the proportion of *Campylobacter* infection to be 2.9% during the dry season as compared to 0% during the rainy season [16]. In contrary studies in Central Africa and Malawi showed a higher prevalence of campylobacter infection during the rainy season [12,13].

### Determinants of *Campylobacter* infections

Campylobacteriosis has been shown to be associated with animals like cattle, goat, pigs and birds like chicken; also with un-boiled water and use of rain water [15,17]. In this study and similar to previous studies [3,15,18], children with acute watery diarrhea living with close proximity to cows were 2.3 times at risk of acquiring campylobacter infections though the difference was not statistically significant. As reported previously [19,20], exposure to inadequately treated water is assumed to be an important risk factor for acquiring *Campylobacter* infection; in this study despite the difference being not statistically significant those children who used un-boiled water had 1.5 times risk of acquiring campylobacteriosis than those used boiled water.

Children co-infected with malaria parasites had a higher risk (3.4 times) of acquiring campylobacter infection than those without co-infection. This co-infection could partly be explained by high prevalence of both diseases in our setting.

### Limitation of the study

This study was a cross-sectional study therefore seasonal variation could not be addressed. The design of the study involved only children with acute watery diarrhea so causal relation could not be confirmed. Also the proportion of eligible children that participated into the study is not known since we did not collect characteristics of these children and their reason for non-participation this might be a source of selection bias. Lastly speciation and drug susceptibility was not done so it is difficult for this study to recommend on treatment policy. Despite these limitations objectives of the study were achieved and discussed.

## Conclusion

*Campylobacter* is present in children with acute watery diarrhea and is more often seen in children attending Sekou Toure Hospital than in BMC. It is associated with an age above 2 years and malarial co-morbidity. We therefore recommend further studies to determine the species of Campylobacter responsible for infection and the susceptibility pattern of the isolate to guide appropriate antibiotic therapy. A large multi-centre study is needed to determine the association of Campylobacter infection with other co-morbidities such as concurrent malaria.

## Competing interests

The authors declare that they have no competing interests.

## Authors’ contributions

AP, MFM, RK and SEM designed the study. DT, MFM, LP, AP, JS and SEM performed culture and microscopy. SEM, AP, JS, BRK, and RK analyzed the data, SEM and MFM wrote the manuscript which was revised and approved by all coauthors.

## References

[B1] Kosek M Bern C Guerrant RL Policy and Practice The global burden of diarrhoeal disease, as estimated from studies published between 1992 and 2000 Bull World Health Organ 2003 72 3 197 204 12764516 PMC2572419

[B2] Coker AO Isokpehi RD Thomas BN Human Campylobacteriosis in developing countries Emerg Infect Dis 2002 72 237 244 10.3201/eid0803.010233 11927019 PMC2732465

[B3] Allos BM Campylobacter jejuni Infections: update on emerging issues and trends Clin Infect Dis 2001 72 8 1201 1206 10.1086/319760 11283810

[B4] Lindblom G Ahren C Changalucha J Gabone R Kaijser B Nilsson L Campylobacter jejuni/coli and Enterotoxigenic Eschericia coli (ETEC) in Faeces from Children and Adults in Tanzania Scand J Infect Dis 1995 72 589 593 10.3109/00365549509047073 8685639

[B5] Rao MR Naficy AB Savarino SJ Abu-Elyazeed R Wierzba TF Peruski LF Pathogenicity and convalescent excretion of Campylobacter in rural Egyptian children Am J Epidemiol 2001 72 2 166 173 10.1093/aje/154.2.166 11447051

[B6] Kish L Survey sampling 1965 New York: A wiley Interscience Publication

[B7] Mshana SE Joloba L Kakooza A Kaddu D Role of microscopic examination of stool specimens in the diagnosis of campylobacter infection from children with acute diarrhoea in Kampala, Uganda Tanzan J Health Res 2010 72 1 0 3 10.4314/thrb.v12i1.56367 20737835

[B8] Kamugisha E Mazigo H Manyama M Rambau P Mirambo M Kataraihya JB Low sensitivity but high specificity of ParaHIT-f in diagnosing malaria among children attending outpatient department in Butimba District Hospital, Mwanza, Tanzania Tanzan J Health Res 2009 72 2 97 99

[B9] Lyamuya EF Aboud S Urassa WK Sufi J Mbwana J Ndugulile F Evaluation of simple rapid HIV assays and development of national rapid HIV test algorithms in Dar es Salaam, Tanzania BMC Infect Dis 2009 72 19 10.1186/1471-2334-9-19 19226452 PMC2650699

[B10] NACP NACP Tanzania National Guidelines For the Management of HIV and AIDS 2012 4 Dar-Es-Salaam: National AIDS Control Program 70 71

[B11] Mshana SE Joloba M Kakooza A Campylobacter spp among Children with acute diarrhea attending Mulago hospital in Kampala - Uganda Afr Health Sci 2009 72 3 201 205 20589152 PMC2887033

[B12] Mandomando IM Macete EV Ruiz J Sanz S Abacassamo F Vallès X Etiology of diarrhea in children younger than 5 years of age admitted in a rural hospital of southern Mozambique Am J Trop Med Hyg 2007 72 3 522 527 17360878

[B13] O’Reilly CE Jaron P Ochieng B Nyaguara A Tate JE Parsons MB Risk factors for death among children less than 5 years old hospitalized with diarrhea in rural western Kenya, 2005–2007 PLoS Med 2012 72 7 e1001256 10.1371/journal.pmed.1001256 22802736 PMC3389023

[B14] Blaser MJ Black RE Duncan DJ Amer J Campylobacter jejuni-specific antibodies are elevated in healthy Bangladeshi children J Clin Microbiol 1985 72 164 167 3972984 10.1128/jcm.21.2.164-167.1985 PMC271606

[B15] Eberhart-phillips J Walker N Garrett N Bell D Sinclair D Rainger W Campylobacteriosis in New Zealand : results of a case–control study J Epidemiol Community Health 1995 72 686 691 10.1136/jech.51.6.686 PMC1060567 9519133

[B16] Casals C Schellenberg D Urassa H Vargas M Gasco J Kahigwa E Etiology of diarrhea in children less than five years of Age Am J Trop Med Hyg 2004 72 5 536 539 15155987

[B17] Tenkate TD Stafford R Risk factors for campylobacter infection in infants and young children: a matched case–control study Epidemiol Infect 2001 72 3 399 404 11811871 10.1017/s0950268801006306 PMC2869763

[B18] Samie A Ramalivhana J Igumbor EO Obi CL Prevalence, haemolytic and haemagglutination activities and antibiotic susceptibility profiles of Campylobacter spp. isolated from human diarrhoeal stools in Vhembe District, South Africa J Health Popul Nutr 2007 72 4 406 413 18402183 PMC2754015

[B19] Newell DG Fearnley C Sources of campylobacter colonization in broiler chickens Appl Environ Microbiol 2003 72 8 4343 4351 10.1128/AEM.69.8.4343-4351.2003 12902214 PMC169125

[B20] Duke LA Breathnach AS Jenkins DR Harkis BA A mixed outbreak of cryptosporidium and campylobacter infection associated with a private water supply Epidemiol Infect 1996 72 303 308 10.1017/S0950268800052614 8666074 PMC2271435

